# Pneumatosis cystoides coli in a patient with stage IV diffuse large B-cell lymphoma

**DOI:** 10.1093/jscr/rjad041

**Published:** 2023-02-10

**Authors:** Gabriel Atan Sanchez, Andrew Michael Thompson

**Affiliations:** Surgical Unit, Wagga Wagga Base Hospital, Wagga Wagga, NSW, Australia; Surgical Unit, Wagga Wagga Base Hospital, Wagga Wagga, NSW, Australia

## Abstract

A 49-year-old female with a background of stage IV diffuse large B-cell lymphoma and subsequent graft-versus-host disease from a bone marrow transplant presented to a rural hospital in New South Wales, Australia with 12-h history of painless per rectal bleeding and fever. On examination she had a soft, but distended abdomen. Laboratory investigations revealed thrombocytopenia and hypokalaemia. Computed tomography of the abdomen and pelvis had a bizarre appearance due to pneumatosis cystoides coli extending from the ileocaecal junction to the mid-transverse colon. Given her benign abdominal examination, her management was initially supportive with intravenous antibiotics, intravenous fluid resuscitation and correction of electrolyte abnormalities.

## INTRODUCTION

Pneumatosis cystoides intestinalis (PCI) is a rare radiological sign that can involve the small intestine and/or the colon. It is characterized by the presence of gas, either in a cystic or linear form within the subserosa or submucosa of the small intestine or colon, respectively [1]. When it affects the colon it is known as pneumatosis cystoides coli. The pathogenesis is poorly understood and is likely multifactorial [2]. Incidence is unknown as most patients are asymptomatic [2].

## CASE REPORT

**Figure 1:**
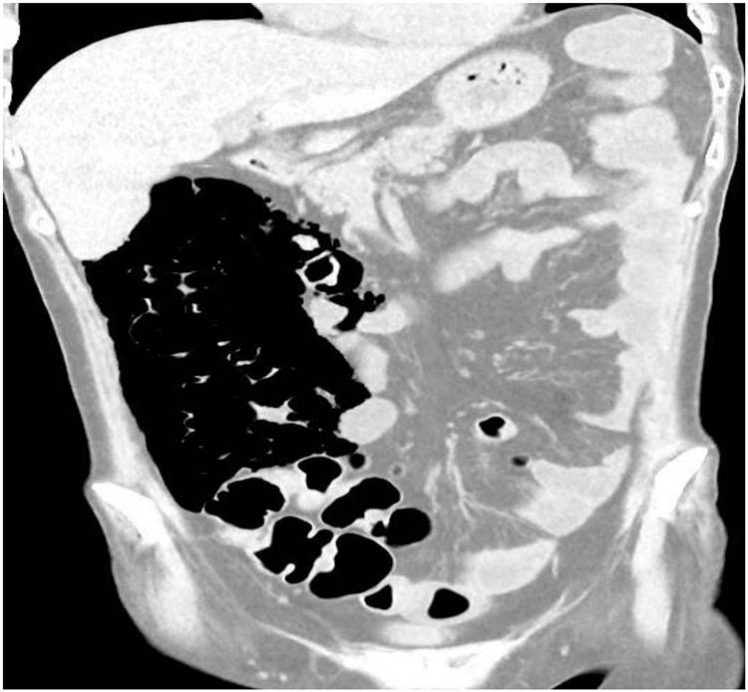
CT scan coronal view.

**Figure 2:**
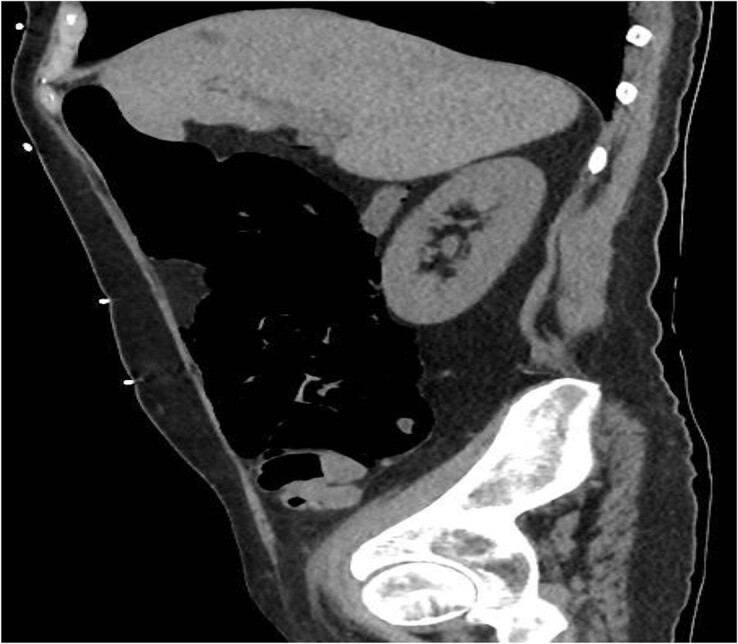
CT sagittal view.

**Figure 3:**
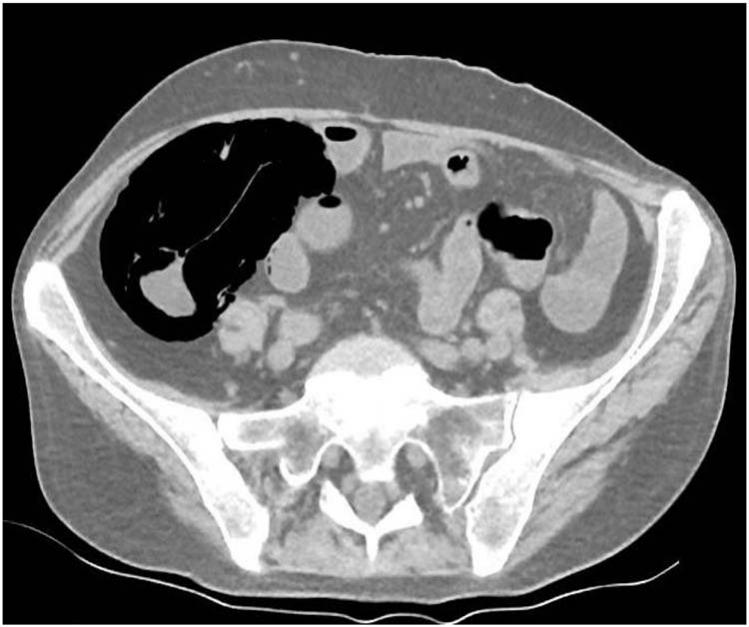
CT axial view.

**Figure 4:**
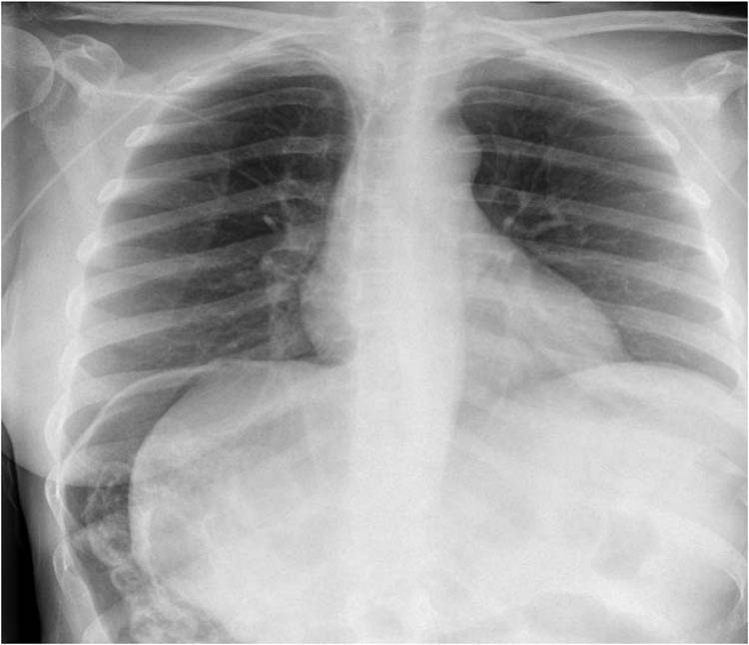
Chest X-ray.

A 49-year-old female with a background of stage IV diffuse large B-cell lymphoma and subsequent chronic graft-versus-host disease (cGVHD) from a bone marrow transplant presented to a rural referral hospital in New South Wales with a 12-h history of painless per rectal bleeding and fever. She had undergone her last cycle of chemotherapy 12 months prior and her cGVHD was being treated with dexamethasone mouthwash, oral cyclosporine and an infusion of rituximab, administered the day prior to this presentation. On examination she had a soft, but distended abdomen. Laboratory investigations revealed thrombocytopenia and hypokalaemia. Computed tomography (CT) of the abdomen and pelvis had a bizarre appearance due to pneumatosis cystoides coli extending from the ileocaecal junction to the mid-transverse colon (Figs 1–3). A chest X-ray was also taken, which shows evidence transmural air within the ascending colon, including the hepatic junction (Fig. 4). Given her benign abdominal examination, her management was initially supportive with intravenous antibiotics, intravenous fluid resuscitation and correction of electrolyte abnormalities. She was subsequently transferred to a metropolitan tertiary hospital, under her usual treating haematologist with consultation by the colorectal surgical team. She recovered and did not require any acute surgical intervention for pneumatosis cystoides coli.

## DISCUSSION

Those with PCI who become clinically apparent present with symptoms related to the PCI itself (abdominal pain, obstruction or per rectal bleeding) or symptoms of the underlying disorder associated with PCI [2]. PCI may be classified as either benign or life-threatening [3, 4]. The former is self-limited and does not require specific treatment. It can be caused by pulmonary disease, systemic disease (scleroderma, AIDS and SLE), intestinal inflammation, iatrogenic injury, long-term corticosteroids (as in this patient) transplant and organ (including bone marrow). Life-threatening PCI requires prompt treatment to reduce morbidity and mortality. This is more likely to be caused by intestinal ischaemia, bowel obstruction, enteritis/colitis, toxic megacolon and collagen vascular disease. The underlying cause should be treated in all these patients, regardless of the presence of symptoms [2]. In the paediatric population, most cases of PCI are secondary to necrotizing enterocolitis, and are more likely to require emergent surgery. Complications occur in ~3% of patients and they include small or large bowel obstruction due to cyst encroachment on the lumen; volvulus; intussusception; adhesions following cyst collapse and ulceration of mucosa overlying the cysts [2].

Take-home messages are as follows:

(1) Pneumatosis cystoides colitis is more commonly associated with patients who are immunocompromised, whether via chemotherapy, radiation therapy, HIV/AIDs or chronic use of corticosteroids [5].
(2) Clinicians should treat the patient and not the CT scan. Though CT findings can appear dramatic, clinically well patients without abdominal signs can be managed non-surgically. A low threshold for intervention should be maintained in the high-risk immunosuppressed cohorts.
(3) In most cases where PCI is diagnosed in infants, it is secondary to necrotizing enterocolitis, usually requiring emergent surgery [2].
(4) The diagnosis of neutropenic enterocolitis (typhlitis) must always be considered as a differential diagnosis in patients who have recently received intensive chemotherapy and have neutropenia on bloods [6, 7]. It is a condition which carries a mortality rate as high as 50% if untreated [8].

## CONFLICT OF INTEREST STATEMENT

None declared.

## FUNDING

None.
